# Densification of Alloying Anodes for High Energy Lithium‐Ion Batteries: Critical Perspective on Inter‐ Versus Intra‐Particle Porosity

**DOI:** 10.1002/advs.202403530

**Published:** 2024-07-08

**Authors:** Yiteng Luo, Yungui Chen, Nikhil Koratkar, Wei Liu

**Affiliations:** ^1^ Institute of New Energy and Low‐Carbon Technology *(INELT)* College of Carbon Neutrality Future Technology Sichuan University Chengdu 610065 China; ^2^ Department of Materials Science and Engineering Rensselaer Polytechnic Institute Troy NY 12180 USA; ^3^ Department of Mechanical Aerospace and Nuclear Engineering Rensselaer Polytechnic Institute Troy NY 12180 USA

**Keywords:** alloying anode, Li‐ion batteries, pore engineering, Si‐C anode

## Abstract

High Li‐storage‐capacity particles such as alloying‐based anodes (Si, Sn, Ge, etc.) are core components for next‐generation Li‐ion batteries (LIBs) but are crippled by their intrinsic volume expansion issues. While pore pre‐plantation represents a mainstream solution, seldom do this strategy fully satisfy the requirements in practical LIBs. One prominent issue is that porous particles reduce electrode density and negate volumetric performance (Wh L^−1^) despite aggressive electrode densification strategies. Moreover, the additional liquid electrolyte dosage resulting from porosity increase is rarely noticed, which has a significant negative impact on cell gravimetric energy density (Wh kg^−1^). Here, the concept of judicious porosity control is introduced to recalibrate existing particle design principles in order to concurrently boost gravimetric and volumetric performance, while also maintaining the battery's cycle life. The critical is emphasized but often neglected role that intraparticle pores play in dictating battery performance, and also highlight the superiority of closed pores over the open pores that are more commonly referred to in the literature. While the analysis and case studies focus on silicon‐carbon composites, the overall conclusions apply to the broad class of alloying anode chemistries.

## Graphical abstract

This perspective quantitatively reveals how the excessive porosity in alloying‐based anodes negates the volumetric and gravimetric energy density of lithium‐ion batteries (LIBs). Judicious porosity control represents a critical criterion to concurrently boost energy metrics and cycle life. Distinguishing intraparticle versus interparticle pores as well as close versus open pores emerges as a critical guide in developing genuine high‐performance LIBs.

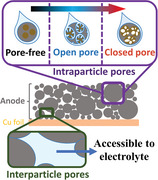

## 1 Introduction

Over the past few decades, lithium‐ion batteries (LIBs) have served as a key enabler for the penetration of renewable energy into human society. However, satisfying the growing demand for both mobile and stationary energy storage remains challenging, despite an annual improvement of 7−8% in energy densities for LIBs technology. The pursuit of higher energy densities (>350 Wh kg^−1^) and lower cost (<100 $/Wh) has provoked continuing endeavors of developing new LIBs materials.^[^ ^1^ ^]^ While the LIBs industry has witnessed progressive advancements on the cathode side, for example the use of high‐nickel Ni‐Co‐Mn/Ni‐Co‐Al layered metal oxide (NCM/NCA) in ≈300 Wh kg^−1^ high energy cells,^[^ ^2^ ^]^ the anode side has remained relatively stagnant. Since the early 1990s when LIBs were first commercialized,^[^ ^3^ ^]^ carbon‐based anodes (graphite, soft/hard carbons) have dominated the prevailing LIB market. Alloying‐based anodes have emerged as the most promising alternative or “drop‐in” to carbon anodes, with the most representative success being silicon due to the exceptionally high capacity of 3579 mA h g^−1^ (based on Li_15_Si_4_),^[^ ^4^ ^]^ a suitable equilibrium potential of 370 mV versus Li^+^/Li and its abundant reserves in Earth's crust.^[^ ^5^ ^]^ These unparalleled advantages render Si‐anode a core component for developing next‐generation LIBs.

The primary impediment to applying alloying‐based anodes in LIBs is the cycle‐induced multidimensional degradation, including cracking/pulverization, unstable solid electrolyte interphase (SEI), and loss of electrical contact, at both the electrode and particle level.^[^ ^6^ ^]^ Taking Si as an example, “Nanosizing” represents a major advancement as the catastrophic particle cracking is found to be largely alleviated by reducing the size of Si to the nanoscale.^[^ ^7^ ^]^ “Compositing”, i.e., embedding Si primary particles into a matrix, represents another avenue to alleviate particle degradation.^[^ ^8^ ^]^ Carbon‐silicon (C‐Si) composites have drawn attention due to their superior electron conductivity, Li‐diffusivity and electrolyte compatibility.^[^ ^8^, ^9^ ^]^ Nonetheless, unlike cathode particles where the Li‐hosting structure undergoes relatively small lattice changes (for instance, ≈6.8% for LFP,^[^ ^10^ ^]^ ≈1.9% for LCO^[^ ^11^ ^]^ and 1–5% for Li(Ni, Co, Mn)O_2_),^[^ ^12^ ^]^ the inescapable ≈380% volume expansion of Si is rooted in the intrinsic density and lattice difference between Si and Li_15_Si_4_.^[^ ^13^ ^]^ The reported stabilization of nano‐ or composite‐based Si‐architectures often relies on “pre‐planting pores” to accommodate the volume expansion in concert with the lithiation reaction. As increasing Si contents in the anode become a mainstream desire, which accesses higher cell energies but brings into play serious volume expansion related problems, astute handling and rationalization of pores represents an urgent task in battery design.

Significant efforts have been devoted to the engineering of Si‐anode pores, highlighting its unique role in determining the cell cycle life and electrode/cell geometrical expansion.^[^ ^1^, ^8^, ^9^, ^14^ ^]^ Amid these endeavors, pore pre‐plantation in Si‐anode represents a prevailing strategy, showing capabilities to extend cycle life and better maintain cell geometry throughout the operation.^[^ ^15^ ^]^ However, the multi‐faceted role of electrode porosity is usually underappreciated: (1) Pores negate cell volumetric energy densities (Wh L^−1^) as they do not store energy but constitute a considerable portion of cell volume. Such porosity‐cell volumetric energy relation explains the general preference for densified particles and electrodes.^[^ ^16^ ^]^ (2) Increase of pore volume would also affect cell gravimetric energy density (Wh kg^−1^) due to the concomitant increase of liquid electrolyte (g Ah^−1^).^[^ ^17^ ^]^ In a typical LIB cell, the electrolyte accounts for ≈ 9.7−16% of the cell weight,^[^ ^18^ ^]^ and ≈9% of the cell cost.^[^ ^19^ ^]^ Therefore reducing the electrolyte amount is an important objective.^[^ ^18^, ^20^ ^]^ (3) Electrode pores can affect the in‐cell stress/strain distribution, being crucial for not only the performance of individual cells but also for the integration of cells into modules and packs.^[^ ^21^ ^]^ For cell types with rigid metal casing, e.g., cylindrical or prismatic cells, a high level of internal stress is a potential safety hazard.^[^ ^22^ ^]^ In pouch cells, the soft packaging permits more swelling but is more restrictive to dimension variations, where approximately expansion of <10% and <25% at the cell and electrode level, respectively, is considered acceptable.^[^ ^9^, ^23^ ^]^

Among all alloying‐based anodes, including Si, Sn, Al, Ge, etc., the volume expansion is proportional to the amount of inserted Li in the host metal during lithiation, roughly following Vegard's law.^[^ ^24^ ^]^ Hence, the introduction of intraparticle pores in high‐capacity alloying‐based anode is deemed necessary and inevitable, either by pore pre‐plantation before cell assembly or by cycle‐induced pore generation during cell operation. there is only very limited recognition in the battery community of how electrode pores impact cell energy metrics and cycle performances. Targeting at >300 Wh/kg, >800 Wh L^−1^ high‐energy LIB cells with reasonable cycle life (>1000 cycles under high depth‐of‐discharge, DOD), the judicious design of pore structures is critical. While over‐extrapolation of LIB energy densities based solely on the weight of active components is known to cause confusion,^[^ ^25^ ^]^ the energy density projection of alloying‐anode‐based cells is even more complex than that of conventional graphite‐based cells due to the more profound impact of electrode porosity. Moreover, despite its criticality, how the volume, distribution, and genre of pores determine the mechanical, electrochemical, and geometrical response of Si‐based electrodes to cycling remains ambiguous. This is rooted in the crucial but poorly recognized impact of ever‐evolving pore structures during cell operation, including Li^+^ diffusion path, reaction kinetics, and stress/strain evolution and distribution. The pursuit of higher cell energies, longer cycle life, and lower cell cost calls for a more judicious design of anode pores. Quantitative analysis and an in‐depth understanding of the unique role of pores offer key and pragmatic value and will guide future R&D directions of alloying‐based LIBs.

While Si‐anode materials have been discussed in prior reviews,^[^ ^8^, ^9^, ^14^, ^25^, ^26^ ^]^ this perspective provides detailed rationale on the density and porosity design of Si‐C based anodes, spanning from the individual particle to the electrode level. The discussion starts with the construction of a single‐stack cell model with practical cell metrics, from which we provide in‐detail pragmatic projections of cell energy metrics with varied anode composition and porosities. We move to the synthetic methodologies that underpin the rationalization and realization of the pore design principles, wherein advanced analytical/diagnostic tools in quantifying or/and monitoring the evolution of pores are also introduced. Finally, future prospects for development are highlighted, providing guidelines for genuinely high‐performance Si‐anodes from a practical viewpoint. While these analysis and case studies focus on Si‐C composites, overall the concepts, methodologies and conclusions apply to the broad class of alloying‐based anode chemistries including Sn, Ge, Al, etc., which are condensed into a tabulated editable form with a built‐in algorithm.

## 2 Impact of Porosity on Cell Energy Projections

To fulfill the stringent requirements of high energy LIBs, maximizing the percentage of active materials and reducing that of inactive materials (i.e., current collectors, electrolytes, conductive agents, and binders, etc.), with respect to overall cell weight/volume, represent a guiding principle. Adopting high active materials mass loading in a densified electrode, namely thick and compact electrodes, is the mainstream approach.^[^ ^1^, ^27^ ^]^ A successful example is using single crystal NCM particles in replacement of polycrystalline analogues. Elimination of intraparticle grain boundaries, defects, and voids, improves the particles’ durability against harsh electrode densifying treatment (calendaring). Without changing anode and cathode chemistries, a denser electrode with lower porosity contributes to boosting the energy density (303 Wh kg^−1^ and 730 Wh L^−1^). Reduced electrolyte dosage also plays a key role in enabling such upgrades.^[^ ^28^ ^]^

In the existing literature, the presence of mesopores and macropores are considered essential elements for stabilizing Si‐anode, ranging from a few nanometers to tens of micrometers.^[^ ^29^ ^]^ As theoretical simulation results have shown that even molecular‐sized pores will allow infiltration of liquid electrolyte,^[^ ^30^ ^]^ it is fair to infer that various pores in the Si‐anode will annex additional liquid electrolyte loadings.^[^ ^31^ ^]^ Increasing the Si‐content or the Si mass loading, will further aggravate such a situation, due to the concomitantly higher pore volumes in the electrode. This arouses critical questions such as: how much electrode pores and liquid electrolytes ought to be introduced with varying Si‐contents? And to what extent does this aspect affect cell volumetric and gravimetric energy densities?

**Figure 1:**
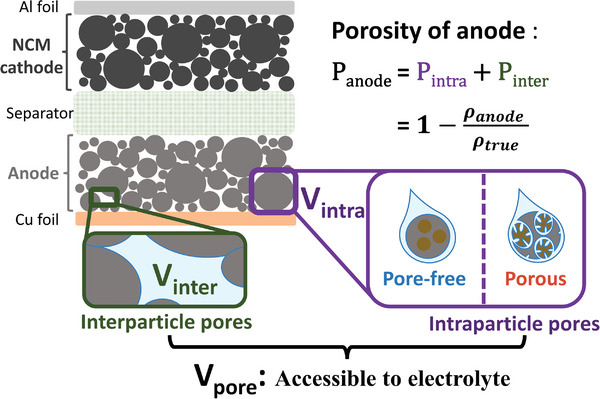
Schematic of a single battery stack as a model system for cell energy projection, where cathode/anode active materials are double‐side coated onto Al and Cu foil current collectors. The impact of anode porosity (P_anode_) on cell energy metrics arises from the varying anode volume (V_anode_) and the associated electrolyte dosage (m_electrolyte_). To enable in‐depth analysis and design of porosity, V_anode_ is deconvoluted into intraparticle porosity (P_intra_) and interparticle porosity (P_inter_).

To quantitatively understand anode‐porosity‐cell‐energy relations, we adopt a single stack cell as a model system that is comprised of double‐sided negative electrodes, a separator, and double‐sided positive electrodes. Such stack energy density, defined as the energy contained in a sandwich of anode, cathode, electrolytes, and separator, allows the high‐fidelity projection of Si‐based LIBs as it can be repeated many times in any cell format. **Figure** **1** represents such a C‐Si||NCM 811 stack battery model. Pairing high‐Ni NCM cathode with Si‐containing anode with thin Cu (≈8 µm) and Al (≈16 µm) current collectors represents a typical cell design. Commercial cell benchmarks were adopted including areal capacity = 4 mAh cm^−2^, NCM cathode porosity of 26% and compaction density of 3.4 g cm^‐3^.^[^ ^32^ ^]^ Liquid electrolytes are to fully infiltrate all existing pores in the anode, cathode, and separator. In order to elucidate how the Si anode impacts cell energy metrics, the anode specifications including Si content and electrode porosity were set as variables, and all other cell specifications remained constant (detailed in Data S1, Supporting Information).

The porosity of anode (*P_anode_*) is defined as the sum of pore volume in the electrode (*V_pore_*) divided by the overall volume of the active coating layer (*V_anode_*):

(1) $$P_{\mathrm{anode}}=\frac{V_{\mathrm{pore}}}{V_{\mathrm{anode}}}$$

The volume and mass of the active coating layer (*V_anode_ and m_anode_*) can be further expressed as follows:

(2) $$V_{anode}=V_{CSi}+V_{CA+B}+V_{pore}$$

(3) $$m_{anode}=m_{CSi}+m_{CA+B}$$

*V_CSi_* and *m_CSi_* are the volume and mass of the C‐Si particles; *V_CA+B_* and *m_CA+B_* are the summed volume and mass of conductive agents and binders (CA+B).

The cell gravimetric and volumetric energy densities (*E_G_* and *E_V_*) are calculated as:

(4) $$E_{G}=\frac{E}{m_{cell}}=\frac{E}{m_{anode}+m_{electrolyte}+m_{else}}$$

(5) $$E_{V}=\frac{E}{V_{cell}}=\frac{E}{V_{anode}+V_{else}}$$

m_cell_ is the summed mass of the cell, which is comprised of the mass of the anode (*m_anode_*), electrolyte (*m_electrolyte_*), and the remaining cell components (*m_else_*, including a cathode, separator, and current collector). *V_cell_* is the summed cell volume and is comprised of the volume of the anode active coating layer (*V_anode_*) and the remaining cell components (*V_else_*) including the cathode and separator.

In the current stack cell, with the anode coating layer being the only variable, *m_else,_* and *V_else_* are constants. The sum of all the pores in the cell represents the amount of required liquid electrolyte, allowing the quantitative analysis of electrolyte loadings (*m_electrolyte_*). Hence *m_electrolyte_* is not a constant but evolves with anode porosity. Empirically, to ensure complete formation of SEI and necessary ion conductance, the volume of electrolyte should be ≈1.5 times the summed volume of all available cell pores,^[^ ^33^ ^]^ and the density of conventional liquid electrolyte (ρ_electrolyte_) is ≈1.2 g cc^−1^.^[^ ^34^ ^]^ Hence, m_electrolyte_ is given as:

(6) $$m_{electrolyte}=1.2\times V_{electrolyte}=1.2\times 1.5\times \left(V_{pore}+V_{rest-pore}\right)$$

*V*_*rest* − *pore*_ refers to the pores in the separator and cathode, being a constant.

As the cell energy (E) is pre‐determined (4 mAh/cm^2^ × cell area × voltage), the cell gravimetric and volumetric density energy (*E_G_* and *E_V_*) is determined by the summed cell weight and volume (*m_cell_* and *V_cell_*). m_cell_ is correlated with m_anode_ and m_electrolyte_, V_cell_ is determined by V_anode_, and m_electrolyte_ is dependent on V_pore_. The above analysis indicates that the anode‐porosity versus cell‐energy relationship hinges on four key variables, that is, m_anode_, m_electrolyte_, V_anode_ and V_pore._

Despite its obvious impact, direct and precise measurement of anode porosity value remains difficult.^[^ ^35^ ^]^ In commercial practice, electrode porosity (P_anode_) is obtained by measuring the electrode compaction density (ρ_anode_, also known as press density):

(7) $$P_{anode}=1-\frac{\rho_{anode}\;}{\rho_{true}}$$

ρ_anode_ (g cm^‐3^) is obtained by the measured mass (g cm^‐2^) of the active coating layer divided by its thickness (cm). ρ_true_ (g cm^‐3^) is the averaged true density of all electrode components including active materials, conductive agents, and binder, being a constant at a given electrode composition.

As shown in Figure 1, unveiling the nature of electrode porosity, one can classify the anode pores into two types: (1) the intraparticle pores that reside inside the particles and this portion of pore volume (*V_intra_*) can only be tuned at the materials synthesis stage. (2) the interparticle pores volume (*V_inter_*) is located in between the particles in the electrode, and can be controlled through electrode calendaring:

(8) $$V_{pore}=V_{inter}+V_{intra}$$

Hence the anode porosity can be further resolved into two:

(9) $$P_{anode}=P_{intra}+P_{inter}=\frac{V_{intra}}{V_{anode}}+\frac{V_{inter}}{V_{anode}}$$

The intraparticle and interparticle porosity (*P_intra_* and *P_inter_*) are defined as the pore volume within the particles or in between the particles divided by the volume of the anode coating layer (V_anode_), respectively.

Taking conventional graphite‐based cells as a baseline, a few generic impacts of incorporating Si into the anode can be envisioned: (1) on one hand, higher anode gravimetric and volumetric capacity of Si compared to graphite (3580 vs 372 mAh g^−1^; 8303^[^ ^18b^ ^]^ versus 841^[^ ^36^ ^]^ mAh cc^−1^) that directly lowers the m_anode_ and V_anode_. This is perhaps the most apparent positive aspect in elevating E_G_ and Ev; (2) on the other hand, after the incorporation of Si the anode porosity P_anode_ needs to be larger than graphite, and the increase of P_anode_ will lead to an increase of m_electrolyte_ and may offset the reduction in m_anode_ (Equation (6)), and the increase in V_pore_ may offset the reduction of V_C‐Si_ (Equation (2)).

**Figure 2:**
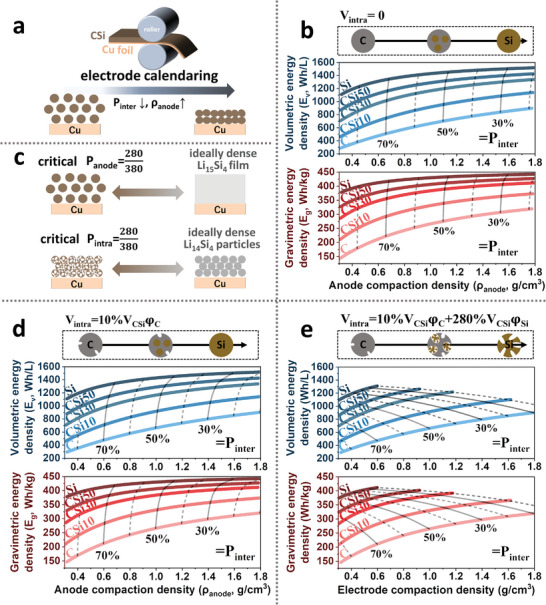
Cell gravimetric and volumetric energy density of C‐Si||NCM 811 cells with varying porosity design. a) Electrode calendaring controls interparticle porosity (P_inter_) and anode compaction density (ρ_anode_). b) Cell energy densities are based on pore‐free C‐Si particles (P_intra_ = 0). c) Assuming ideally compact film or particle at the lithiated state points to the critical anode porosity (P_anode_) and intraparticle porosity (P_intra_) at the de‐lithiated state, respectively. d) Cell energy densities adopting porous C‐Si particles with the intraparticle pore volume being a function of carbon content (φ_C_). e) Cell energy densities adopting porous C‐Si particles with the intraparticle volume being a function of the content of both carbon and silicon (φ_C_, φ_Si_).

**Figure** **2**a illustrates the control of anode compaction density (ρ_anode_) and porosity (P_anode_) via electrode calendaring. In practical LIBs, the graphite negative electrode is subject to proper calendaring until compaction densities of ρ_anode_ =   ≈ 1.6 g cm − ^3^, corresponding to P_anode_ of ≈26% (P_intra_+ P_inter_).^[^ ^37^ ^]^ For commercial graphite (P_intra_≈0), the P_inter_ of 20% can be considered as a benchmark that achieves proper electric contact and Li‐ion diffusion.^[^ ^38^ ^]^ Hence the porosity‐cell‐energy analysis can proceed based on three typical C‐Si particles with varying P_intra_ set‐up.

### 2.1 Pore‐Free C‐Si Particles (V_intra_ = 0, V_pore_ = V_inter_)

Figure 2b depicts the gravimetric and volumetric energy densities (E_g_ and E_v_) of C‐Si systems with pore‐free dense particles, that is P_intra_ = 0, and P_anode_ = P_inter_ (V_intra_ = 0, and V_pore_ = V_inter_). Various Si contents ranging from 0, 10, 30, 50, and 100 wt% Si were considered, termed C, CSi10, CSi30, CSi50, and Si respectively. Electrode compaction density (ρ_anode_) was plotted as X‐axis ranging from 0.3 to 1.8 g cm^−3^, corresponding to P_inter_ ranging from ≈80% to ≈20%. One may see that the volumetric energy increases linearly with the increase of ρ_anode_ due to the reduction of electrode volume (V_anode_). To one's surprise, the gain in gravimetric energy density is also quite profound as ρ_anode_ increases. For instance, CSi10 system shows an increase from 237 to 365 Wh/kg when ρ_anode_ increases from 0.4 to 1.6 g/cm^3^. This is directly correlated with the reduction of electrolytes due to the reduction of anode pore volume (V_pore_). At any Si‐content, the E_g_ shows significant upward trends as ρ_anode_ increases. This gain in E_g_ can be understood as “squeezing” the inactive electrolyte out of the system.

At ρ_anode_ levels of 1.1 g cm^−3^ (approximately corresponds to P_inter_ = 50%), incorporation of a small amount (10 wt%) Si leads to very substantial improvements in both E_g_ and E_v_ as compared to that of the graphite‐based (pure C) baseline (953 vs 698 Wh L^−1^ and 338 vs 274 Wh kg^−1^). However, such gain in E_g_ and E_v_ becomes less significant when further increasing the Si content in high‐Si systems. For instance, increasing from 50 wt% to 100 wt% gives 1316 versus 1450 Wh L^−1^ and 410 versus 432 Wh kg^−1^ improvements. This justifies the widespread endeavor toward developing C‐Si systems with < 30 wt% Si. We highlight that baseline graphite‐based cells, with a typical ρ_anode_ of 1.6 g cm^−3^, possess 309 Wh kg^−1^ and 852 Wh L^−1^. To achieve on‐par E_g_ or E_v_ using CSi10 anode, the ρ_anode_ needs to be >0.79 or >0.87 g cm^−3^, respectively. This highlights the importance of stringent control over ρ_anode_ of Si‐containing systems in the hope of positive gain as compared to the graphite baseline.

Unlike the typical ceramic cathodes where particle density remains almost constant at charged/discharged states, the anode side is deemed to experience considerable volume change. For example, going from C_6_ to LiC_6_ generates ≈10% of expansion.^[^ ^39^ ^]^ C‐Si composites will experience even higher volume change.^[^ ^23b^ ^]^ In fact, even carbonaceous active particles (artificial graphite, natural graphite, meso‐carbon microbeads, etc.,) are never ideally pore‐free. A certain amount of intraparticle pore volume (V_intra_) always exists as grain boundaries, defects, and voids in the battery‐grade particles. These void spaces are formed at the material synthesis stage, for instance, granulation and pitch coating steps.^[^ ^40^ ^]^ For artificial graphite, the >2500 °C high‐temperature treatment for graphitization leads to contraction of carbon precursor and leaves considerable V_intra_.^[^ ^41^ ^]^ These pores served as a beneficial factor in maintaining structural stability towards extended cycle life,^[^ ^42^ ^]^ rendering the intraparticle porosity (P_intra_) necessary.

The intrinsic volume change of active C‐Si particles, which is rooted in the crystallographic lattice changes of C‐to‐LiC_6_ and Si‐to‐Li_15_Si_4_ alloying reactions, leads to the rationalization of critical P_anode_ and P_intra_ values. This is demonstrated in Figure 2c. Taking pure Si anode as an example, assuming the ideally compact Li_22_Si_5_ film at the lithiated (charged) state, leads to the critical P_anode_ = 280/380 of porous Si at the de‐lithiated state. Similarly, assuming the ideally dense Li_22_Si_5_ particles at the charged state, the critical P_intra_ is 280/380. The critical P_anode_ and P_intra_ largely determine the **minimal amount of porosity** that is required in the as‐fabricated electrode or particles to fully accommodate the volume change. If P_anode_ and P_intra_ were initially below these critical values, one may expect the P_anode_ and P_intra_ to continue to grow during cycling, as such cycle‐induced porosity increase is well established.^[^ ^43^ ^]^ This necessitates the astute design of P_intra_ and pre‐plantation of intraparticle pore volume (V_intra_).

### 2.2 Porous C + Dense Si (V_intra_ = 10%V_CSi_φ_C_, V_inter_ = V_pore_ ‐V_intra_)

Introducing Si primary particles into a porous carbon host represents a prevailing C‐Si secondary particle design, showing encouraging progress.^[^ ^23^, ^44^ ^]^ The astute design of P_intra_ (and V_intra_) of such C‐Si particles should be based on the volume fraction of carbon and Si (φ_*C*_ and φ_*Si*_) and the overall volume of the C‐Si particles (**V**_**CSi**_). Here, in replacement of ideally dense C‐Si particles, introducing a certain amount of intraparticle pore volume (V_intra_) in accordance with the volume fraction of carbon (V_intra_ = 10% V_CSi_φ_C_) results in cell energy densities that are shown in Figure 2d. There is an overt decrease in Ev due to the introduction of V_intra_. For example, under the same P_inter_ = 20%, the compaction density of pure C (ρ_anode_) has dropped from 1.76 to 1.60 g cm^−3^, leading to Ev dropping from 890 to 851 Wh L^−1^. One may see the curves of each C‐Si material show a similar shape in Figure 2d and in Figure 2b. This is because introducing intraparticle pores (V_intra_) is in essence the redistribution of the overall V_pore_: part of the pores being moved from in between the particles into the inner space of C‐Si particles. This change in principle provides better volume accommodation for each particle, benefitting cycle performances. A successful case in this regard is the creation of pores in graphite (P_intra_ of graphite up to 23%) followed by the incorporation of chemical vapor deposition (CVD) grown Si into these pores.^[^ ^45^ ^]^ The resulting C‐Si anode‐based pouch cells present an improvement of 12.6% in E_g_ and 15.9% in E_v_ as compared to graphite‐based cells.^[^ ^45a^ ^]^

As the “porous C + dense Si” model (V_intra_ = 10% V_CSi_φ_C_) captures the volume expansion of carbon, a few critical problems emerge: (1) the volume expansion of Si‐to‐Li_15_Si_4_ is much more concerning than C‐to‐LiC_6_ transition; (2) the increase of Si content (decrease of C‐content, φ_C_) will decrease the intraparticle volume (V_intra_), which can lead to an increasingly higher risk of failed particle structure at high Si contents. For instance, one may even observe a slight increase of compaction density (ρ_anode_) if increasing the Si content: at P_inter_ = 20%, the pure C electrode shows ρ_anode_ of 1.6 g cm^−3^ while the CSi50 shows ρ_anode_ of 1.7 g cm^−3^. Such an increase of ρ_anode_ is associated with the decrease of V_intra_, both are inappropriate. Hence we underline that the “porous C + dense Si” model is limited to low‐Si content systems, e.g., <20 vol% Si. For high‐Si content particles, cycle‐induced pressure fluctuation inside the C‐Si particles can cause particle and electrode degradation.^[^ ^14^, ^46^ ^]^ More careful design of V_intra_ is therefore needed.

### 2.3 Porous C + Porous Si (V_intra_ = 10%V_CSi_φ_C_+280%V_CSi_φ_Si_, V_inter_ = V_pore_‐V_intra_)

Ideally, V_intra_ should be a function of the composition of C‐Si as the volume accommodation needs are proportional to the Si and C content. Adopting a “porous C + porous Si” based V_intra_ design (V_intra_ = 10% V_CSi_φ_C_ + 280% V_CSi_φ_Si_), V_intra_ can represent the required pore volume to fully “digest” the lithiation‐induced expansion of both carbon and Si components. This may be interpreted as incorporating porous Si primary particles into a porous carbon host. Ideally, such porous C‐Si particles will transform into dense Li_15_Si_4_+LiC_6_ particles upon lithiation. Figure 2e depicts the projection of cells E_g_ and E_v_ under such porosity design.

Compared to the curves shown in Figure 2d, a noticeable change in Figure 2e is that the highest achievable ρ_anode_ significantly decreased. This trend is more overt in high‐Si systems. For instance, with a neat Si system, shifting from pore‐free Si to porous Si (from Figure 2d,e), the electrode compaction density decreases from 1.82 to 0.48 g cm^−3^ at identical P_inter_ = 20%. From the topmost curve in Figure 2e (neat Si), we highlight that the highest ρ_anode_ is 0.6 g cm^−3^, corresponding to the highest energy density of 411 Wh kg^−1^ and 1308 Wh L^−1^ achieved with ideal porosity (P_anode_ = P_intra_ = 280/380 and P_inter_ = 0). Here this ρ_anode_ reflects the intrinsic porosity requirement of Si, as one cannot expect a pore‐free Si electrode to cycle in a reversible manner. Hence the projection in Figure 2e presents the **highest accessible E_g_ and E_v_ of C‐Si systems with the most ideal porosity design**.

We note that the space utilization rate of hexagonal close‐packed (HCP) sphere assemblies is 74%, respectively (summed volume of spheres/overall volume). This highlights the intrinsic interparticle porosity in a powder‐based electrode with granular particle constituents. Considering the proper granulometric distribution contributes a slightly higher space‐filling, i.e., small spheres filling the interspace of larger spheres, it is feasible to assume a baseline P_inter_ of 20% in a powder‐based electrode. Considering a P_inter_ = 20%, electrode compaction densities (ρ_anode_) become 1.60, 1.30, 0.94, 0.74, and 0.48 g/cm^3^ for C (graphite), CSi10 CSi10, CSi30, CSi50, and Si, respectively, coinciding well with the values in commercial electrodes.^[^ ^37^, ^47^ ^]^ It should also be noted that as Si content increases above CSi30, the increase of E_v_ and E_g_ appears to be less pronounced. For instance, at P_inter_ = 30%, the C, CSi10, CSi30, CSi50, and Si systems displayed E_g_ of 297, 340, 379, 382, 394 Wh kg^−1^, and E_v_ of 796, 964, 1142, 1147, 1201 Wh L^−1^, respectively. This suggests that the positive gains in E_g_ and E_v_ by incorporating a higher amount of porous‐Si are being increasingly compromised by the accompanying increase of electrolyte amount and pore volume. This in principle represents the top ceiling of energy densities of Si‐based cells.

### 2.4 From Open to Closed Pores

The aforementioned results and discussion highlight the profound impact of anode porosity. In general, the pores that are needed to fully digest Si expansion greatly compromise the E_G_ by increasing electrolyte weight. A most appealing porosity design should be the introduction of pores but without a concomitant increase of liquid electrolytes. The key of this “advanced” porosity design is the isolation of V_intra_ from the liquid electrolyte. In poreology, all material pores can be classified into two categories according to their accessibility to the surrounding environment: closed pores and open pores. Closed pores refer to the ones that are isolated from the surroundings, while open pores on the contrary could communicate with external substances, including the adsorption and permeability of molecules (for instance N_2_, I_2,_ and mercury).^[^ ^48^ ^]^

**Figure 3:**
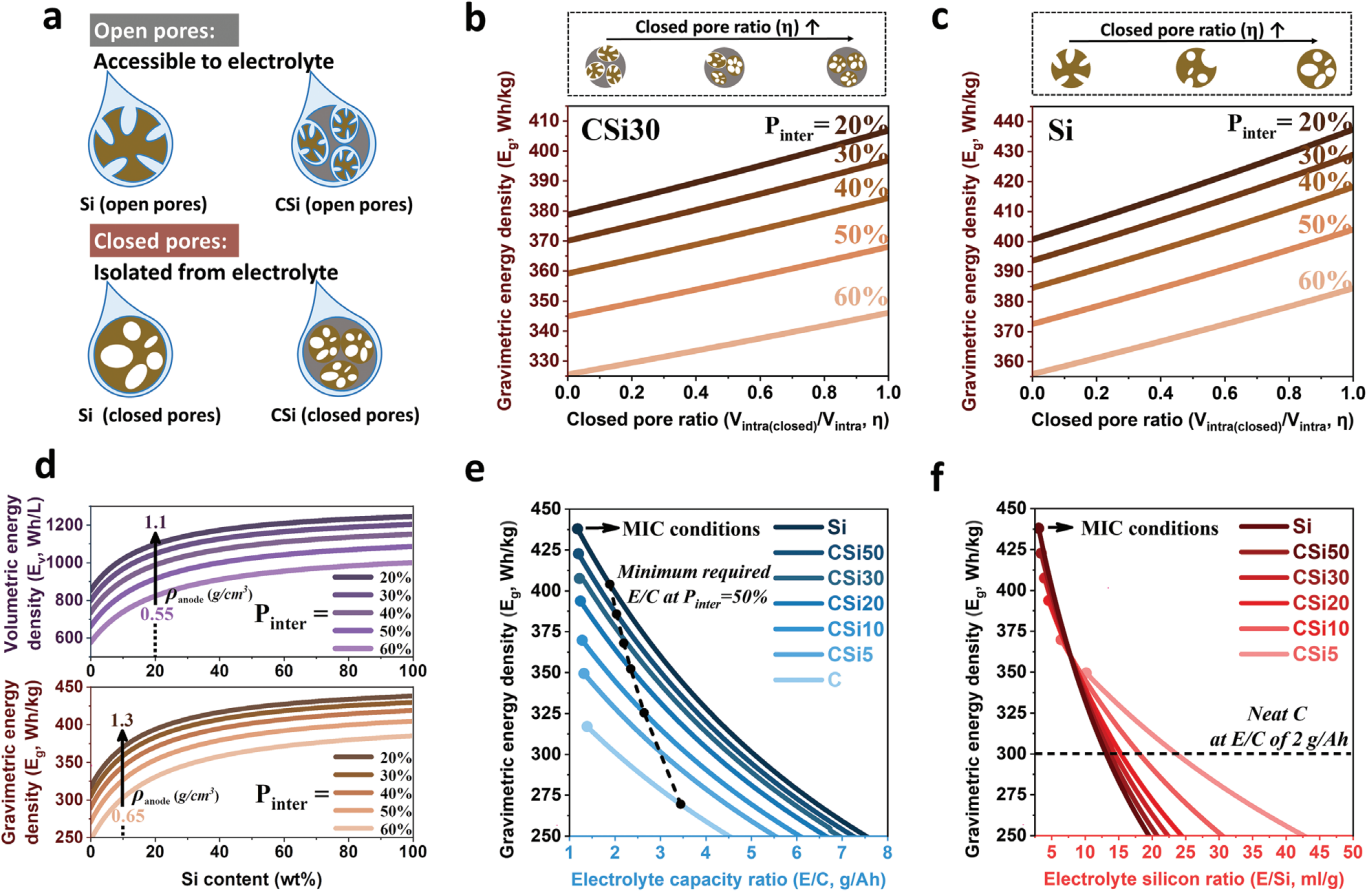
The cell energy density of C‐Si||NCM 811 cells, where the C‐Si particles are pre‐planted with intraparticle pores (10% V_Csi_φ_C_ + 280% V_Csi_φ_Si_). a) the accessibility to electrolyte classifies two main intraparticle pore types: open pores that will be filled by electrolyte, and closed pores that are isolated from the electrolyte. b, c) Cell energy densities of C‐Si||NCM 811 cells at 30 wt% Si (b) and 100 wt% Si (c) with varying closed pore ratio (η = V_intra(closed)_/V_intra_) at fixed optimal P_intra_. d) Cell energy densities at various Si contents and P_inter_ at ideal η = 1.0. e) Cell gravimetric energy densities under various electrolyte capacity ratios (E/C), the minimum required E/C corresponds to the most ideal condition (*MIC*). f) Cell gravimetric energy densities under various electrolyte silicon ratios (E/Si), the minimum required E/Si corresponds to *MIC*.

Borrowing from this classic concept, an advanced pore design may be proposed as turning the intraparticle pores to be “closed” pores. As shown in **Figure** **3**a, this transition enables the introduction of V_intra_ without the concomitant increase of electrolyte amount. We introduce a closed pore ratio (η) that quantitatively captures such transition: 0 corresponds to fully open intraparticle pores and 1.0 corresponds to completely closed intraparticle pores. Herein the required amount of electrolyte can be expressed as:

(10) $$\begin{array}{ccc} m_{\mathrm{electrolyte}} & = & \rho_{\mathrm{electrolyte}}V_{\mathrm{electrolyte}}=1.2\times 1.5\\ & & \times \;\left[V_{\mathrm{inter}}+\left(1-\eta \right)V_{\mathrm{intra}}+V_{\mathrm{rest}-\mathrm{pore}}\right] \end{array}$$

Comparing Equation (6) and Equation (10), one may see that closing the intraparticle pores would reduce the amount of electrolyte, giving rise to positive gains in E_g_. As shown in Figure 3b, all the E_g_ curves of CSi30 increase almost linearly with the increase of the closed pore ratio. For instance, at P_inter_ = 30%, transforming V_intra_ from open to closed pores gives rise to E_g_ increase from 379 to 407 Wh kg^−1^. This E_g_ gain is equivalent to adding approximately >70% more additional Si into the anode (i.e., shifting CSi30 to pure Si). We note that the positive effect of closed pores becomes even more profound at high Si contents. As Figure 3c shows, for pure Si at P_inter_ = 20%, an increase of η from 0 to 1 leads to an increase of E_g_ from 401 to 437 Wh kg^−1^, pointing out a highly practical path toward >400 Wh kg^−1^ cells. Comparing this E_g_ value to those shown in Figure 2, one may notice that this (E_g_ = 437 Wh kg^−1^) represents by far the highest E_g_ achieved in the current model, which is achieved when the following three key conditions are satisfied:

(11) $$\begin{array}{c} \left\{\begin{array}{c} P_{\mathrm{inter}}=20\%\\ V_{\mathrm{intra}}=10\%\;V_{\mathrm{CSi}}\varphi_{C}+280\%\;V_{\mathrm{Csi}}\varphi_{\mathrm{Si}}\\ \eta =1 \end{array}\right.\left(\mathrm{most}\;\mathrm{ideal}\;\mathrm{condition},\;\mathrm{MIC}\right) \end{array}$$

To achieve such optimal conditions, the first equation calls for very harsh/aggressive calendaring of the electrode, the second requires precise control of intraparticle porosity in concert with the particle composition, and the last requires an ideal coating layer on the CSi particles that can completely shield off the liquid electrolyte. **When all three aforementioned conditions are satisfied, one may expect a most stable and genuine high‐energy C‐Si particle structure**.

Therefore, closed pores over open pores are strongly advocated for designing C‐Si particles. With a closed pore ratio of η = 1, one can expect that no extra electrolyte is needed when increasing Si‐content in the anode. Hence such a scenario represents the utmost E_g_ achievable with C‐Si anode, which can be envisioned in Figure 3d. The maximum E_g_ and E_v_ of 100% Si anode for harshly calendared electrodes (i.e., delivering P_inter_ = 20%) can reach 437 Wh/kg and 1244 Wh/L. At this ideal circumstance, calendaring‐based compacting of CSi10 electrode from 0.65 to 1.3 g cm^−3^ could gain E_g_ from 302 to 369 Wh kg^−1^, and E_V_ from 736 to 1017 Wh L^−1^. In fact, the E_g_ and E_v_ values can reach up to 407 Wh kg^−1^ and 1142 Wh L^−1^ (at P_inter_ = 20%) with CSi30. Further increase in Si content is less appealing considering limited further gains in E_g_ and E_v_ coupled with reduced cost‐effectiveness and higher engineering difficulties. This leads to the conclusion that C‐Si particles with <30% Si will be a judicious choice.

These conclusions point to the key impact of electrolyte dosage levels that are strongly correlated with pores of C‐Si electrodes. In commercial practice, the electrolyte capacity ratio (E/C, in g/Ah) is more often used to quantify the electrolyte dosage levels.^[^ ^20^, ^33^ ^]^ The cell energy density under various E/C ratios can be projected and shown in Figure 3e, with the ***MIC*** at the left side point representing the minimum electrolyte amount of 1.2–1.4 g Ah^−1^ for each C‐Si system, which coincides with the practical value of ≲2 g Ah^−1^ for commercial evaluation.^[^ ^49^ ^]^ Maintaining a low E/C ratio contributes to the high E_g_ levels with various Si contents. For instance, controlling E/C at 2 g Ah^−1^ will contribute to an E_g_ of 301, 328, 344, 364, 378, 387, and 400 Wh kg^−1^ for C (graphite), CSi5, CSi10, CSi20, CSi30, CSi50, and Si. And to achieve >300 Wh kg^−1^ cells, E/C levels need to be lower than 2, 3, 3.6, 4.2, 4.5, 4.8, and 5 g Ah^−1^ for these C‐Si systems.

One may see that the parameter of E/C (g/Ah) is indifferent to the Si content, hence being unable to capture the different amounts of required liquid electrolytes among systems with varying Si content. Hence, the electrolyte‐Si ratio (E/Si, in ml g^−1^) can be regarded as a useful criterion that reflects the efficacy and genuine stability of Si‐based anode. This parameter is somewhat analogous to the concept of E/S ratio in Li‐S batteries.^[^ ^50^ ^]^ E/Si considers the competitive impact between the increasing amount of Si and the increases of electrolyte amount in authentically determining the attainable gravimetric energy density (E_g_). High‐Si loading systems with good cycle life but under unrealistically high E/Si would lead to decreases of cell energy (E_g_) compared to the graphite baseline. Choosing ***MIC*** as the left side point, the electrolyte‐Si ratio (E/Si) with corresponding energy density is shown in Figure 3f. For example, controlling E/Si ratio lower than 7.8 ml g_si_ will ideally contribute to an E_g_ >360 Wh kg^−1^ for all Si‐containing systems, corresponding to diverse E/C ratios lower than 1.6, 2.1, 2.4, 2.7 and 3 g Ah^−1^ for CSi10, CSi20, CSi30, CSi50 and Si. If adding >5 wt% Si to achieve an E_g_ of 300 Wh/kg, < 23 ml g_si_^−1^ E/Si ratio is required, which reduces to < 10 ml g_si_^−1^ for 350 Wh kg^−1^, and to < 5 ml g_si_^−1^ for 400 Wh kg^−1^. We also highlight that with low Si content, it is possible to achieve E_g_ higher than high‐Si systems by minimizing E/Si ratio. This may be achieved by closing the intraparticle pores (η closer to 1) and lowering interparticle voids (i.e., lower P_inter_).

As intraparticle closed pore are expected to possess key advantages in avoiding electrolyte flood, resulting in lithiation‐delithiation process arguably depicted as inward breathing.^[^ ^51^ ^]^ However, how the closed pores affect Li‐ion diffusion pathway and lithiation/de‐lithiation kinetics is yet to be uncovered. While one may conjecture that closed pores as opposed to open pores may have sluggish Li‐diffusion in the particle interior, due to its absence of electrolyte contact with interior parts, a recent study has in fact demonstrated that intraparticle closed pores act as a Li reservoir sites, thereby facilitating the diffusion of lithium.^[^ ^52^ ^]^ However the impact of closed pores on Li‐diffusivity is correlated with the dimension/geometry of pores, the properties of their surrounding (pores’ matrix), and the radii of secondary particles. Moreover, one may view the pore‐Li‐diffusion relation as a dynamic process, as during cycling the pores dimension/geometry can evolve thus changing the intraparticle Li‐diffusion pathways.^[^ ^14^, ^35^ ^]^ Although from a practical point of view, a C‐Si composite with closed pores is in general more favorable over open‐pores due to the low‐SSA, high‐TD and low E/Si ratio, the understanding of the Li^+^ storage/diffusion process in closed/open pores, as they might differ greatly, remains rather unclear. An in‐depth understanding of this issue can better connect with battery performances, including capacity, rate and cycle life etc., but requires development of advanced tools that we will discuss in the next section.

This pores‐closing concept is applicable in all‐alloying‐type anodes including but not limited to C‐Si, encompassing Sn, Ge, Al, Sb‐based systems that exhibit severe volume expansion when serving as Li/Na/K storage host. Although numerous studies have revealed the critical role of pore design in the hope of gaining sufficient cycle life,^[^ ^53^ ^]^ it remains challenging to accurately evaluate the cell energy densities when these alloying‐based anode materials are used. We note that the concept and methodology of ***MIC*** presented here can be further extended to any alloying‐type anodes. **Hence, these holistic considerations for connecting anode porosity (both inter‐particle as well as intra‐particle) to authentic cell energy metrics are summarized in an editable form with a built‐in algorithm (supplied as Supporting Information)**. It is worth noticing that this form applies to all alloying‐type anodes, providing pragmatic projections of cell energies that sheds light on designing pore structures towards authentic high energy cells.

## 3 Engineering and Characterization of Particle Pores

The profound influence of pores on cell energy densities highlights the unique and critical value of controlling porosity and pore structure. While the interparticle pores are mainly dictated by electrode calendaring, the intraparticle pores are highly tunable and require special attention at the materials synthesis stage. Ideal intraparticle pores should combine three important attributes: (1) accommodating expansion to mitigate particle cracking and electrode swelling; (2) mechanically robust to endure harsh calendaring and prolonged cycling; (3) closed pores to shield off liquid electrolyte (liquid‐tight). These aspects apply to not only Si‐based but also all alloying‐based anode particles. A deeper understanding of the impact of not only pore size and volume but also their geometry and distribution is pivotal. Using Si as the most representative case, here we provide a key perspective on guidelines for engineering the particle pores and for accurately characterizing the obtained pore structures.

### 3.1 Synthetic Engineering of Pores

**Figure 4:**
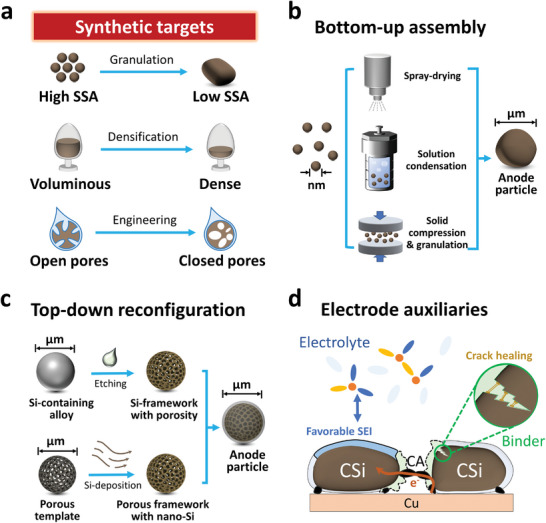
Synthetic pathways for achieving dense alloying‐based anode particles with engineered in‐built porosity. a) The main objective of particle engineering encompasses three main targets: low specific surface area (SSA), high density, and closed intraparticle pores. b) Dense particles can be achieved by adopting a bottom‐up assembly strategy, where nano‐Si and other ingredients (such as carbon precursors) aggregate into microparticles in the spray solution or dry state. c) The top‐down approach involves re‐configuration of micron‐sized precursor particles, where the nano‐porous Si‐framework or porous framework with Si decoration is a critical intermediate. d) Novel inactive electrode auxiliaries including electrolytes, binders, and conductive agents that operate in concert with the volume changes during cell operation, can significantly boost the longevity of high‐capacity alloying anodes.

Towards a genuine well‐performing practical Si‐anode, synthetic methods for dense Si‐based particles with well‐engineered pores are urgently needed and in the following section, we present some promising approaches in this regard. In the particle scale, the main objective encompasses three main targets: low specific surface area (SSA), high density, and high closed pore ratio (**Figure** **4**a), where the electrolyte‐proofing surface layer constitutes the core component. Various surface coating strategies were found to create isolation from the electrolyte for Si‐based secondary particles (tabulated in **Table** **1**). We emphasize that analogous strategies should be effective for other alloying‐type anode particles. Aside from the surface coating layer, the genuinely stable electrochemical performance hinges to mitigated structural degradation over cycling, involving two aspects: (1) densifying the pore‐containing Si‐based particles to achieve inherent structural stabilities; (2) applying electrode auxiliary components that adapt and harmonize with the dense closed‐pore particles.

**Table 1:** Various surface coatings for constructing closed‐pore C‐Si composites.

Type	Work	SSA [M^2^ G^−1^]		ICE	
		Pristine	Coated	Pristine	Coated
PYROLYTIC CARBON	[60]	32.4	8.1	68.7%	84.3%
	[59]	228.0	3.3	78.6%	82.0%
	[14d]	972.0	8.8	/	/
	[14b]	9.4	1.4	/	90.9%
	[61]	/	/	80.3%	87.8%
	[62]	120.0	12.6	86.6%	80.3%
CVD CARBON	[63]	/	/	81.4%	87.4%
	[64]	544.0	257.0	/	84.0%
	[29e]	229.0	/	37.6%	88.7%
	[45a]	/	5.2	90.0%	92.0%
	[65]	104.1	61.5	/	75.0%
POLYMER	[66]	13.5	10.8	84.4%	84.6%
	[67]	42.1	13.7	84.6%	81.3%
	[68]	/	/	88.0%	90.0%
	[69]	94.8	5.7	77.3%	84.4%

#### 3.1.1 Bottom‐Up Particle Assembly

Whereas nanosizing Si represents a major strategy toward a stabilized particle structure, nanosizing‐related drawbacks arise due to the dramatic increase of specific surface area and decrease in tap density.^[^ ^8^, ^54^ ^]^ Challenges arise in restraining surface side reactions, constructing stable electrical contacts, and lowering the anode porosity to reduce electrolyte dosage. Assembling Si nanoparticles (SiNPs) into micro‐sized secondary particles represents one of the most useful designs, which is schematized in Figure 4b. For instance, spray drying is widely adopted in industrial powder production featuring high output and cost‐effectiveness.^[^ ^55^ ^]^ It can also be used for the assembly of C‐Si secondary granules, where the process often involves the co‐dispersion of SiNPs and carbon precursor into a liquid media, followed by spray‐drying such slurry to form composite granules.^[^ ^56^ ^]^ An alternative strategy involves high temperature and high‐pressure treatment in a hydro‐/solvothermal reactor, where the thermo‐condensation reaction of specific components in the mixture, like polymers or pitch, leads to the formation of dense granular particle precursors.^[^ ^57^ ^]^ Another alternative strategy involves the mechanical fusion process where the deformation of soft components such as pitch allows the granulation of the Si‐containing mixture, enabling solvent‐free high output production.^[^ ^58^ ^]^ These granulated particles then were subject to further pyrolysis to yield C‐Si composite particles. The evaporation of solvents or volatile components during spay drying, or the pyrolysis of the soft template (components that decompose to gaseous species with little solid leftover) are the key elements in controlling the porosity of C‐Si secondary particles. Hence, the concentration, distribution, and assembly of these pore‐forming agents are critical controlling points. A densification treatment on the assemblies of SiNPs and carbon precursors under varying mechanical forces can also be utilized to tune the pore structure in the final C‐Si structure.^[^ ^59^ ^]^

#### 3.1.2 Top‐Down Particle Reconfiguration

As bottom‐up assembly made prominent advances towards densifying Si‐anode, a shared feature of these approaches is the use of nano‐sized Si primary particles (SiNPs) as building blocks. Nonetheless, the production of SiNPs hinges on a complex synthesis process, e.g., prolonged sand milling of Si or chemical vapor deposition (CVD) of Si‐containing gas (SiH_4_, SiCl_4_). The limited production efficiency and high manufacturing cost of SiNPs poses severe challenges for large‐scale application. Alternatively, the top‐down particle reconfiguration approach represents emerging opportunities to produce porous Si with nano‐features, exempt from the need to obtain SiNPs (Figure 4c). This approach mainly involves (1) the fabrication of micro‐sized porous Si skeleton; (2) coating or filling porous Si with carbon. The size of the obtained particles will remain in the range of microns, while the encapsulated Si possesses nano‐architecture and behaves analogous to SiNPs. Compared to the bottom‐up assembly approaches, the top‐down synthesis approach often involves the creation of porous Si, either by etch‐off secondary components from a Si‐containing alloy or by depositing Si into a porous micro‐sized template. This avoids the difficulties in the fabrication and dispersion of the SiNP powder. While this method reduces the fabrication complexity, caution needs to be exercised with the use of dangerous etchants (like HF) or Si‐containing gases (like SiH_4_) as the cost and potential environmental hazards impose great challenges for large‐scale adoption.

#### 3.1.3 Symphonic Electrode Auxiliaries

Aside from structural changes inside the particles, the electrochemical performance of electrodes is often heavily affected by particle‐to‐particle interactions and interactions between active and inactive auxiliary components, including conductive agents (CAs), binders, and electrolytes (Figure 4d).^[^ ^70^, ^71^, ^72^, ^73^, ^74^, ^75^, ^76^, ^77^, ^78^, ^79^, ^80^, ^81^, ^82^, ^83^, ^84^, ^85^, ^86^, ^87^, ^88^, ^89^, ^90^, ^91^, ^92^, ^93^, ^94^, ^95^, ^96^, ^97^, ^98^, ^99^, ^100^, ^101^ ^]^ Numerous studies reveal that Si‐particle electrochemical behaviors can be tuned through the use of novel binders, CAs, and electrolytes. An institutive strategy is the adoption of advanced auxiliary components that can better interact with Si surfaces, i.e., better Si‐binders or Si‐CAs bonding. The electrolyte chemistry also plays a key role in stabilizing Si‐particles by forming stable SEI layers and suppressing side reactions.^[^ ^71^ ^]^ There are several ongoing efforts in developing novel electrolytes for stabilizing Si micro‐particles (SiMPs), which is a key enabler for high tap density electrodes.^[^ ^14^, ^49^ ^]^ Aside from binders and CAs, advanced current collectors,^[^ ^72^ ^]^ or separators,^[^ ^73^ ^]^ or solid‐state electrolytes^[^ ^26^, ^74^ ^]^ can all contribute in this regard.

#### 3.1.4 Future Synthetic Development for Pore Engineering

In summary, as intraparticle pores originate during materials synthesis, the materials precursors and synthetic processes together play a decisive role in dictating the engineering of such pores. We highlight that micro‐sized particles and thick electrodes are prevalent desires, therefore granulation and densification of Si‐containing particles represent the mainstream approach. In this context, the manipulation of intraparticle pores is crucial and highly skillful for stabilizing alloying electrodes and extending electrochemical cycle life. However, achieving a homogeneous distribution of pores with appropriate size and geometry in concert with the neighboring Si‐active phases remains a challenge. In this regard, vapor‐grown Si into a porous carbon host has garnered considerable attention including a few industry start‐ups, and is expected to find niche applications in the future.^[^ ^75^ ^]^ Despite the synthetic difficulties, the key concept behind the particle pore engineering is the isolation of electrolyte from reaching the particle core and the prevention of electrochemical particle‐cracking and Si‐sintering. Realization of this concept hinges on conformal and closed pore structures colocalizing with the Si primary particles, where the Si‐volume changes are fully buffered without incurring electrolyte flooding. The key scientific questions that must be addressed include: How do pores affect the Li‐diffusion path thus impacting lithiation homogeneity and kinetics inside the secondary particles?; How to decouple the pore‐to‐pore and Si‐pore interactions during cell operation?; and how to engineer the pore structures into desirable sizes, morphologies, and distributions in a green, scalable, and cost‐effective manner? These questions are interrelated and extremely complex in the context of practical battery cells. Answers to these questions will constitute the basis for future synthetic routes that produce alloying anodes with optimized porosity and performance.

### 3.2 Characterization and Modeling of Pores

Powder‐based tap density (TD) and electrode‐based compaction density (CD) can be viewed as an indirect and semi‐quantitative means to describe electrode porosity. Nonetheless, this method is incapable of resolving the location and electrolyte‐accessibility of the pores, i.e., distinguishing intraparticle pores from interparticle pores, and closed pores from open pores. Extensive efforts have been devoted to developing advanced characterization tools to precisely capture the structure and evolution of particle pores in alloying anodes. Characterization methods that can quantify the pore structure both at the material powder state and/or at the electrode bulk state are emerging key enablers for the next stage of development. **Table** **2** tabulates the main tools for the characterization of pores, which could be divided into three categories: microscopy, gas/fluid intrusion, and spectroscopy.

**Table 2:** Characterization methods for quantifying pores in particles or electrodes.

Methods	Function	Pore Types
SEM/FIB‐SEM	Electron contrast (surface) Allows in situ set‐ups^[^ ^52a^ ^]^	Probe depth: a few microns Spatial resolution (scale): >10 nm^[^ ^76^ ^]^
TEM	Electron contrast (bulk) Allows in situ set‐ups	Probe depth: thin specimens (< 150 nm) Spatial resolution (scale): atomic scale^[^ ^81^ ^]^
AFM	Mechanical feedback (surface)^[^ ^78^ ^]^	Probe depth: Surficial pores Spatial resolution (scale): atomic scale
Xrm/X‐CT	X‐ray adsorption contrast^[^ ^79^ ^]^	Probe depth: Full‐scale depth Spatial resolution (scale): ∼102 nm (X‐ray beam size)
NMR	Spin‐lattice relaxation of fluids contained in solid pores^[^ ^80^ ^]^	Probe depth: a couple of millimeters Spatial resolution (scale): > 100 µm[81]
MIP	Mercury to assess porosity, average pore diameter, and SSA^[^ ^82^ ^]^	Probe depth: full range (open pores) Spatial resolution (scale): NA
LNA	N_2_ adsorption to assess porosity, pore shape, size distribution, and SSA	Probe depth: full range (open pores) Spatial resolution: NA
SAXS, SANS	Electron and neutron density contrast^[^ ^83^ ^]^	Probe depth: >millimeters Spatial resolution (scale): 0.1∼104 nm

#### 3.2.1 Microscopy for Pore Visualization

Characterization methods that can quantify the pore structure both at the material powder state and/or at the electrode bulk state are emerging key enablers for the next stage of development. Table 2 tabulates the main tools for the characterization of pores, which could be divided into three categories: microscopy, gas/fluid intrusion, and spectroscopy.

Microscopy including scanning electron microscopy (SEM),^[^ ^35a^ ^]^ transmission electron microscopy (TEM),^[^ ^35b,c^ ^]^ and atomic force microscopy (AFM)^[^ ^78^ ^]^ can capture the morphology of pores in a semi‐quantitative manner based on contrast or feedback from the electron beam or a cantilever. However, these microscopy approaches for pore characterization can only characterize the pores on the particle surface (SEM, AFM) or inner pores in thin species (TEM) due to the intrinsic penetration capability of the electron beam (few microns at maximum). To overcome the probe depth limits, an Ar^+^‐based ionic mill or Ga^+^‐based focused ion beam (FIB)^[^ ^84^ ^]^ can be employed to obtain specimens’ cross‐section that allows the visualization of intraparticle pores. However, due to the local heating of ion beam,^[^ ^85^ ^]^ FIB may introduce artifacts that call for further sample pre‐treatment or instrumental infrastructure upgrades such as cryogenic set‐ups.^[^ ^86^ ^]^ In general, electron‐beam‐based microscopy offers powerful spatial resolution down to the nanometer scale, however, it can only provide limited probe depth (a few microns). In contrast, X‐ray can provide nearly unlimited probe depth to distinguish pores from solid substances in a non‐destructive manner due to the sharp adsorption contrast. X‐ray microscopy (XRM) and computed tomography (X‐CT) can offer 2D and 3D images over large specimen dimensions, albeit with limited spatial resolution due to the X‐ray beam size (hundreds of microns). With X‐ray sources such as synchrotron radiation and an advanced charge‐coupled device (CCD) detector, the spatial resolution may be upgraded to ≈100 nm (Nano‐CT) with high time resolution that allows electrochemical operando monitoring.^[^ ^87^ ^]^

#### 3.2.2 Intrusion and Spectroscopy for Pore Quantification

Unlike microscopy, intrusion and spectroscopy‐based analysis is built upon statistically averaged information, thus allowing the comprehensive quantification of pores in specimens with large dimensions. Fluid or gas intrusion methods can be used to directly measure the open pores, including mercury intrusion porosimeter (MIP) and liquid nitrogen adsorption (LNA),^[^ ^29^, ^88^ ^]^ however they are unable to detect closed pores that are inaccessible to the fluids or gases absorbents. To study the closed pores, spectroscopic methods including small‐angle X‐ray scattering (SAXS),^[^ ^35^, ^77^ ^]^ and small‐angle neutron scattering (SANS) can be employed, utilizing the electron or neutron density contrasts between pores and solids.^[^ ^89^ ^]^ SANS possesses higher probe depth for determining pore structures, owing to the larger wavelength and penetration depth of neutrons as compared to X‐ray. The formation of SEI films and the evolution of pores under cycling can also be captured by SANS.^[^ ^90^ ^]^ Similarly, nuclear magnetic resonance (NMR) can acquire quantitative information about pores based on spin‐lattice relaxation of the labeled molecules that are confined in the pores, i.e., H‐containing molecules and hyperpolarized xenon to yield H‐ and ^129^Xe‐ spectrum.^[^ ^80^, ^91^ ^]^ We highlight here that the combination of intrusion and spectroscopy offers a pathway to differentiate open/closed pores from overall particle porosity, thus determining the closed pore ratio (η).

#### 3.2.3 Modeling of Dynamic Porosity

Extensive efforts have been devoted to developing theoretical underpinnings and advanced characterization tools to capture the structure and evolution of particle pores in alloying anodes. Understanding the mechanical response of C‐Si particles and pores is challenging due to the difficulties in quantifying the properties of pores and Li_x_Si alloys under different Li‐concentrations, i.e., different state of charge (SOC). Moreover, the anisotropic lithiation of crystalline Si further complicate this issue,^[^ ^92^ ^]^ i.e., the Li‐diffusion and reaction phase boundary will preferentially propagate along the zone axis of <110>.^[^ ^92^, ^93^ ^]^ Modeling and simulation studies provide unique frameworks for the analysis of these issues.

The modeling framework usually involves a number of structural factors of the C‐Si particles, spanning from the size, distribution, geometry and stoichiometry of both the pores and the C and Si components. Previous studies combine density functional theory (DFT) with molecular dynamics (MD) to capture the Li‐ion transport process and correlate that with plastic deformation of Si, and agree reasonably well with in situ measured lithiation‐induced softening in thin Si‐films.^[^ ^94^ ^]^ However, MD or DFT based simulation are often limited to small‐sizes systems with tens to hundreds of atoms. Though benefiting from the fast development of new algorithms (or even artificial intelligence) and high‐performances supercomputers, the scope of these methods can now be expanded to nanoscale, but conducting analysis over larger pore structures is a challenge. Finite element method (FEM) considers an infinite dimensional system (body) where the position of a typical particle (element) is defined by means of a vector. A dimensional reduction of the target system can be achieved by reducing the admissible motions of the body.^[^ ^95^ ^]^ For modeling anode materials with closed pores, FEM offers unique advantages in integrating smaller regions to a larger system, and the volume expansion and deformation of Si caused by lithiation can be modeled in a way that mimics the thermal expansion using a J2‐flow theory.^[^ ^45^, ^95^, ^96^ ^]^ We note that mechanical deformation and chemical lithiation at the particle or electrode scale can be coupled through FEM, elucidating the dynamic nature of porosity.^[^ ^97^ ^]^

#### 3.2.4 Future Development in Simulation and Diagnosis on Pores

As the size, distribution, and morphology of pores are key aspects to achieving energy‐dense and stable Si‐anodes, quantitatively predicting and characterizing the pores and monitoring their evolution in the context of battery cells cannot be overemphasized. Whereas modeling and simulations provide theoretical analysis on the stress evolution and distribution of the inside of the C‐Si particles, the working environments of active particles in real battery cells are far more complex than in simulation models. Mechanical responses of nano‐sized particulates, often adopted as FEM elements, differs enormously from that of the ones in the practical cells.^[^ ^93^, ^97^ ^]^ Simulation methods that considers the cell conditions, such as real‐time changes in concentration polarization, voltage polarization, Li‐diffusion coefficient, Poisson's ratio, etc., have been reported.^[^ ^95^, ^98^ ^]^ However holistic integration of all these elements are highly challenging. With the growing computational power and AI‐assisted capabilities, developing advanced simulations tools can play an increasingly important role.

Advanced in situ analytical and diagnostic tools, with higher time resolution,^[^ ^99^ ^]^ or spatial resolution^[^ ^100^ ^]^ are of keen interest so that the real‐time evolution of pores can be tracked with precision during the lithiation and de‐lithiation process. However, one may argue that the working environment in in situ cells differs substantially from that in practical cells. For instance, to enable real‐time monitoring of active particles with ultrahigh spatial resolution, electrochemical in situ TEM operates under a high vacuum with two metal‐wire probes and omits the impact of electrolyte‐derived SEI. Environmental in situ TEM amends this issue by adopting non‐volatile ionic liquid electrolytes and/or air‐tight liquid cells with an electron‐transparent window, however, it compromises spatial resolution. Operando analysis of practical cells adopting high‐penetration probes such as X‐ray and neutron beam, can capture pore evolution under working conditions. However, accurate measurements of the quantity and structure of intraparticle pores, in a way that can differentiate closed pores from open pores, and volume‐accommodating‐effective pores from non‐effective ones, is challenging.

In situ observation of the evolution behavior of intraparticle closed pores (e.g., splitting, growth, coalescence, or collapse) is a challenging but much needed task. The real‐time study of pore evolution can significantly alter our current understanding of pore functionality and provide invaluable guidance for the design of pores as well as secondary particles. Though existing studies have claimed that interconnected pores can serve as channels to boost Li‐ion transportation,^[^ ^101^ ^]^ we note that it lacks rigorous expounding in a way that differentiates the Li‐transport acceleration due to increases in electrolyte contact surface area or due to changes in pore interconnectedness. This further highlights the importance of quantifying the closed pore ratio (η). Furthermore, only with advanced in situ analytical tools, can one obtain an accurate dynamical portrayal of the interactions among the intraparticle pores, including aggregation and merging of pores and potential electrolyte invasion, as well as the proliferation and propagation of pores. Further innovation in this aspect should be a major focus for future endeavor.

## 4 Concluding Remarks

In summary, while developing next‐generation LIBs hinges on high‐capacity alloying‐based particles, the intrinsic accompanying volume expansion represents a major challenge. Taking Si‐based anode particles as a typical case, the impact of pore structures inside or in between the secondary particles has been revisited. We highlight the crucial role of differentiating “intraparticle” pores versus “interparticle” pores and “open” pores versus “closed” pores. We discussed how pore engineering (i.e., pore size, volume, geometry, and their spatial distribution) impacts cell energy density, both volumetric and gravimetric, highlighting the importance of quantifying and controlling electrode pore structures. Exciting opportunities emerge as our analysis indicates that the performance of LIBs was dictated by controlling the pore structure of the Si‐based anode, which appears to be more overriding than the Si per se. Various synthetic strategies, as well as characterization tools, were overviewed that can effectively visualize and quantify pores at the particle and electrode levels. Unsettled scientific and technological issues on pore engineering are discussed to point out directions that are most likely to benefit future LIB development. Through continued efforts, pore engineering can and will play a pivotal role in developing genuine high‐performance anode particles. In particular, alloying anode particles with a dual personality –, i.e., dense as a whole but with a controlled distribution of “closed” pores on the inside, represents the best prospects for continued improvements in the gravimetric and volumetric energy density of LIBs.

## Conflict of Interest

The authors declare no conflict of interest.

## Author Contributions

Y. L. and W. L. performed conceptualization; Y. L., Y. C. and W. L. performed methodology; Y. L. and W. L. performed investigation. Y. L. wrote the original draft. N. K. and W. L. wrote review & edited the original draft; N. K. and W. L. performed funding acquisition. N. K. and W. L. performed supervision.

## Supporting information

Supporting Information
