# Supplementary material for: Densification of Alloying Anodes for High Energy Lithium‐Ion Batteries: Critical Perspective on Inter‐ Versus Intra‐Particle Porosity
Source: Adv Sci (Weinh). 2024 Jul 8;11(34):2403530. doi: 10.1002/advs.202403530 (PMC11425885; doi:10.1002/advs.202403530)
Supplement: Supplementary file 1 — Supporting Information [file ADVS-11-2403530-s001.docx]

**Supplementary information for**

Densification of alloying anodes for high energy lithium-ion batteries: critical perspective on inter- vs. intra-particle porosity

Yiteng Luo^a^, Yungui Chen^a^, Nikhil Koratkar^b,c^*, Wei Liu^a**^

a: Institute of New Energy and Low-Carbon Technology (INELT), College of Carbon Neutrality Future Technology, Sichuan University, Chengdu, China

b: Department of Materials Science and Engineering, Rensselaer Polytechnic Institute, Troy, NY, USA

c: Department of Mechanical, Aerospace and Nuclear Engineering, Rensselaer Polytechnic Institute, Troy, NY, USA

**Email:**

*Nikhil Koratkar, koratn@rpi.edu;

**Wei Liu, [weiliu@scu.edu.cn](mailto:weiliu@scu.edu.cn)

Supplemental Information includes one table.

**Data S1**. Critical parameters and built-in algorithm in alloying-anode||NCM 811 cells. (**Double-click on table to allow editing with built-in Excel**.)
